# Injurious Effects of Emodin on Maturation of Mouse Oocytes, Fertilization and Fetal Development via Apoptosis

**DOI:** 10.3390/ijms131113911

**Published:** 2012-10-29

**Authors:** Mei-Hui Chang, Shao-Chung Chang, Wen-Hsiung Chan

**Affiliations:** Department of Bioscience Technology and Center for Nanotechnology, Chung Yuan Christian University, Chung Li 32023, Taiwan; E-Mails: heather740713@yahoo.com.tw (M.-H.C.); hero-steven@yahoo.com.tw (S.-C.C.)

**Keywords:** emodin, apoptosis, oocyte maturation, embryonic development

## Abstract

Emodin (1,3,8-trihydroxy-6-methylanthraquinone), a major constituent of rhubarb, has a wide range of therapeutic applications. Previous studies have established that emodin induces apoptosis in the inner cell mass and trophectoderm of mouse blastocysts and leads to decreased embryonic development and viability, indicating a role as an injury risk factor for normal embryonic development. However, the mechanisms underlying its hazardous effects have yet to be characterized. In the current study, we further investigated the effects of emodin on oocyte maturation and subsequent pre- and post-implantation development, both *in vitro* and *in vivo*. Notably, emodin induced a significant reduction in the rates of oocyte maturation, fertilization, and *in vitro* embryonic development. Treatment of oocytes with emodin during *in vitro* maturation (IVM) led to increased resorption of postimplantation embryos and decreased fetal weight. Experiments using an *in vivo* mouse model disclosed that consumption of drinking water containing 20–40 μM emodin led to decreased oocyte maturation and *in vitro* fertilization, as well as early embryonic developmental injury. Notably, pretreatment with a caspase-3-specific inhibitor effectively prevented emodin-triggered injury effects, suggesting that impairment of embryo development occurs via a caspase-dependent apoptotic process.

## 1. Introduction

Emodin (1,3,8-trihydroxy-6-methylanthraquinone), one of the major chemical constituents of the root of rhubarb (*Rheum palmatum* L.), is widely used in the Orient [1], and exerts immunosuppressive, anticancer, antiinflammatory, antiatherosclerotic, and vasorelaxant effects [2–5]. Emodin inhibits cell proliferation in different cancer cell lines, including HER-2/neu-overexpressing breast cancer [6], hepatoma [7], leukemia [8], and lung cancer [9]. An earlier study reported that emodin-stimulated apoptosis is mediated via reactive oxygen species (ROS) and mitochondria-dependent pathways in human tongue squamous cancer SCC-4 cells [10]. Interestingly, emodin exerts both cytotoxic and protective effects in rat C6 glioma cells [11]. Moreover, recent experiments by our group showed that emodin induces a decrease in mouse embryonic development and viability *in vitro* and *in vivo*[12]. Interestingly, the hazardous effects of emodin on embryonic development were effectively prevented by caspase-9 and -3 inhibitors [12], implying that emodin triggers apoptosis of mouse blastocysts, leading to impairment of embryo development through intrinsic apoptotic pathways. Accordingly, we propose that emodin is an injury risk factor for normal embryonic development in mouse blastocysts.

Oocyte viability is affected by the microenvironment during growth and maturation. Heat stress, oxygen concentration, and glucose content are key determinants of oocyte viability [13–15]. A number of researchers have investigated the influence of environmental biological toxins on oocyte maturation *in vivo* and *in vitro*. During normal embryogenesis, apoptosis (a unique morphological pattern of cell death) functions to remove abnormal or redundant cells in preimplantation embryos [16,17]. However, apoptotic processes do not occur prior to the blastocyst stage during normal mouse embryonic development [18], and induction of cell death during oocyte maturation and early stages of embryogenesis (*i.e.*, via exposure to a teratogen) leads to developmental injury [14,19–22]. While we have established that emodin promotes cell apoptosis and developmental injury in blastocyst-stage embryos [12], the influence of this compound on early-stage embryogenesis processes, such as oocyte maturation, fertilization, and sequential embryo development from zygotes, is currently unclear. Here, we focused on ascertaining whether emodin has a hazardous effect on oocyte development. Briefly, oocytes were incubated with emodin for 24 h and sequential development compared with that of oocytes under emodin-free conditions, with the aim of determining whether short-term exposure to the compound at the oocyte stage has a long-term injurious impact on embryo development. Our results clearly demonstrate that emodin exposure during the oocyte stage not only inhibits oocyte maturation but also promotes injurious effects on *in vitro* fertilization and embryonic development.

## 2. Results

### 2.1. Effects of Emodin on Oocyte Maturation Status, Fertilization Rate, and *in Vitro* Embryo Development

**Figure 1:**
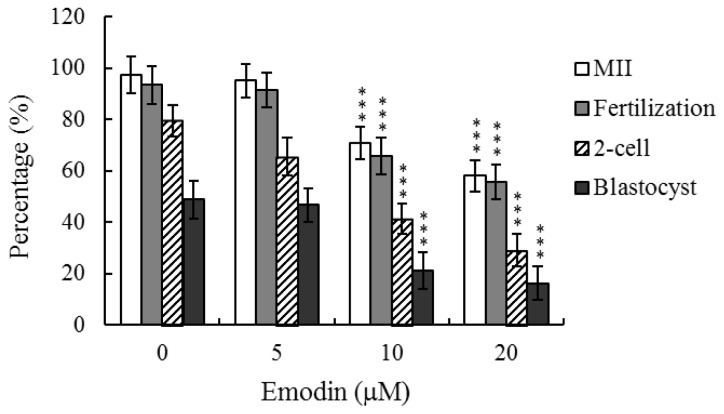
Effects of emodin on mouse oocyte maturation and embryo development *in vitro*. Oocytes were collected from 21 day-old mice, cultured for 24 h in *in vitro* maturation (IVM) medium containing emodin (5, 10 or 20 μM), fertilized *in vitro*, and transferred to *in vitro* culture (IVC) medium. Oocyte maturation, *in vitro* fertilization, cleavage and blastocyst development were analyzed. Values are presented as means ± SD of eight determinations. Data are based on 250–280 samples per group. *** *p* < 0.001 *versus* the untreated control group.

While emodin evidently induces apoptosis and developmental injury in mouse blastocysts [12], its effects on oocyte maturation have not been clarified to date. Oocyte nuclear maturation status was measured using eight independent experimental replicates, with ~250 oocytes per group. The number of oocytes that reached the metaphase II (MII) stage of maturation after *in vitro* maturation (IVM) ranged to about 97%. A lower maturation rate was observed in the emodin-treated oocyte group, which was dose-dependent (Figure 1). Male pronucleus formation was assessed for the detection of fertilization. Our data showed that the ability of oocytes to be fertilized by fresh sperm was significantly decreased upon pretreatment with emodin, prior to IVM (Figure 1).

We further analyzed *in vitro* embryo development to the two-cell and blastocyst stages. Emodin pretreatment led to a significant decrease in cleavage of oocytes to the two-cell stage, indicative of an injurious effect (Figure 1). In addition, the number of embryos cleaved to form blastocysts in the emodin-treated groups was markedly lower than that in untreated control groups (Figure 1).

### 2.2. Effects of Emodin on Cell Proliferation and Apoptosis of Embryos during Oocyte Maturation *in Vitro*

**Figure 2:**
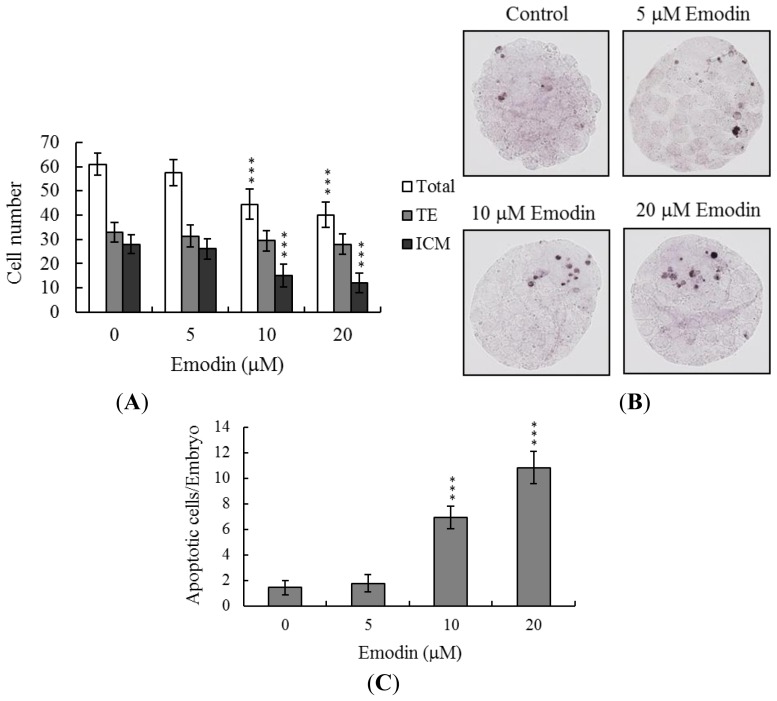
Effects of emodin on cell number and apoptosis in embryos during IVM of oocytes. Oocytes were cultured for 24 h in IVM medium containing emodin (5, 10 or 20 μM), fertilized *in vitro*, and transferred to *in vitro* culture (IVC) medium for *in vitro* development. (**A**) Cell numbers of total, trophectoderm (TE) lineages and inner cell mass (ICM) were counted in blastocysts. (**B**) Apoptotic cells were examined at the blastocyst stage using TUNEL staining, followed by light microscopy. Positive cells are depicted in black. (**C**) The mean number of apoptotic (TUNEL-positive) cells per blastocyst was calculated. Values are presented as means ± SD of six determinations. Data are based on at least 200 samples in each group. *** *p* < 0.001 *versus* the untreated control group.

Total blastocyst cell numbers were determined following emodin treatment during IVM of oocytes, with a view to establishing its effects on cell proliferation, assessed using differential staining followed by cell counting. Significantly lower blastocyst cell numbers were derived from emodin-pretreated oocytes, compared to control oocytes (Figure 2A). Additionally, the numbers of inner cell mass (ICM) cells in blastocysts during IVM were decreased upon emodin pretreatment, while, in contrast, trophectoderm (TE) cell numbers were not affected (Figure 2A).

Apoptosis of blastocysts derived from emodin-pretreated oocytes was additionally evaluated. TUNEL staining revealed a dose-dependent increase in apoptosis of blastocysts from the emodin-pretreated oocyte group (Figure 2B). Further quantitative analysis showed a 7- to 10-fold increase in apoptotic blastocysts derived from emodin-pretreated oocytes, compared to the control group (Figure 2C).

### 2.3. Developmental Potential of Blastocysts from Oocytes Treated with Emodin and *in Vivo* Effects of Emodin Intake on Oocyte Development

**Figure 3:**
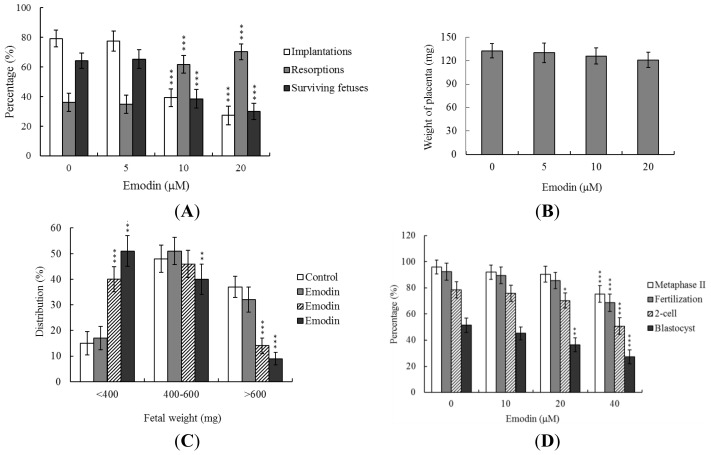
Effects of emodin treatment or dietary emodin intake on embryo development during oocyte IVM. Oocytes were cultured for 24 h in IVM medium containing emodin (5, 10 or 20 μM), fertilized *in vitro*, and transferred to *in vitro* culture medium for development. (**A**) Implantation, resorption and surviving fetuses were analyzed, as described in Materials and Methods. The implantation percentage represents the number of implantations per number of transferred embryos × 100. The percentage of resorption or surviving fetuses represents the number of resorptions or surviving fetuses per number of implantations × 100. (**B**) Placental weights of 40 recipient mice were measured. (**C**) Weight distribution of surviving fetuses at 14 days post-transfer (18-day fetuses). Surviving fetuses were obtained via embryo transfer of control and emodin-pretreated groups, as described in Materials and Methods (320 total blastocysts across 40 recipients). (**D**) Random female mice (21 days old) were fed a standard diet and drinking water supplemented with emodin (10–40 μM) for 5 days or left untreated. Oocytes were collected for *in vitro* maturation, *in vitro* fertilization, cleavage, and blastocyst development analyses. Data are based on at least 300 samples in each group. * *p* < 0.05, ** *p* < 0.01 and *** *p* < 0.001 *versus* the emodin-free group.

Embryos were transferred to 45 recipients per group (8 per horn). A total of 40 recipients were pregnant in at least one horn at day 18. The implantation ratio of blastocysts derived from the oocyte group treated with 20 μM emodin during IVM was ~27%, which was significantly lower than that observed for control blastocysts (~79%) (Figure 3A).

Embryos that implanted but failed to develop were subsequently resorbed in the uterus. The proportion of implanted embryos that failed to develop normally was significantly higher in the 20 μM emodin-treated group (~70%), compared to the control group (~36%). Moreover, the emodin-pretreated group displayed a higher resorption rate than the untreated control group (Figure 3A). In terms of embryo survival rate (surviving fetuses), 64% of the control group survived to 14 days post-transfer (18-day fetuses), compared to only 30% of the 20 μM emodin-treated group (Figure 3A). Interestingly, however, the placental weights of blastocysts derived from emodin-treated oocytes in IVM were not significantly different from those of the control group (Figure 3B). Importantly, fetal weights were lower in the groups treated with 10–20 μM emodin, relative to the untreated control group. Furthermore, only 9% of fetuses in the 20 μM emodin-pretreated group weighed over 600 mg, whereas 37% of control fetuses exceeded this threshold, an important indicator of successful embryonic and fetal development (Figure 3C). Our findings collectively indicate that exposure of oocytes to emodin during IVM reduces the potential of postimplantation development.

In view of the injurious effects of emodin on oocyte maturation and embryo development *in vitro*, we analyzed emodin activity *in vivo* via intake in an animal model. Female mice were fed a standard diet and drinking water supplemented with emodin (10–40 μM) for 4 days, or left untreated, prior to COC collection. Oocyte maturation status, fertilization rate, and *in vitro* embryo development were evaluated. Dietary emodin induced a significant decrease in oocyte maturation and fertilization, resulting in inhibition of embryonic development from the zygote to blastocyst stage (Figure 3D).

### 2.4. Effects of Emodin Intake on the Developmental Potential of Blastocyst-Stage Embryos

**Figure 4:**
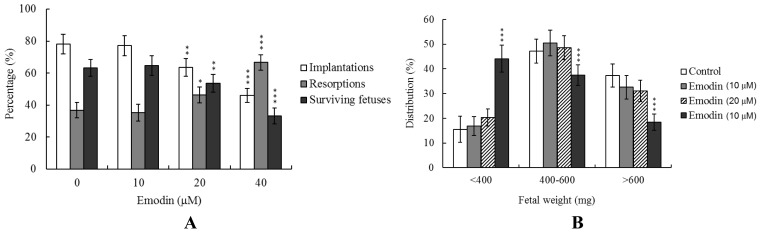
Effects of dietary emodin on embryo development in mouse blastocysts. Random female mice (21 days old) were fed a standard diet and drinking water continuously supplemented with emodin (10–40 μM) or left untreated for 4 days before embryo transfer to the uterus during the experimental period. (**A**) Implantation, resorption and surviving fetuses were analyzed, as described in Materials and Methods. The implantation percentage represents the number of implantations per number of transferred embryos × 100. The percentage of resorption or surviving fetuses represents the number of resorptions or surviving fetuses per number of implantations × 100. (**B**) Weight distribution of surviving fetuses at day 18 post-coitus. Surviving fetuses were obtained by embryo transfer of control and emodin intake groups, as described in Materials and Methods (320 total blastocysts across 40 recipients). * *p* < 0.05, ** *p* < 0.01 and *** *p* < 0.001 *versus* the emodin-free group.

To further determine the effects of emodin on embryo implantation and post-implantation development, we analyzed its *in vivo* activity via intake and transfer of blastocyst-stage embryos to the uterus horn using the embryo transfer assay in an animal model. Female mice were fed a standard diet and drinking water continuously supplemented with emodin (10–40 μM) or left untreated for 4 days before embryo transfer to the uterus during the experimental period. Embryos were transferred to 50 recipients per group (8 per horn). A total of 40 recipients were pregnant at day 18. Notably, the implantation ratio of blastocysts in the emodin intake group was significantly lower than that of control blastocysts (Figure 4A). Moreover, the emodin intake group displayed a higher overall resorption rate than the emodin-free group (Figure 4A). The embryo survival rate of the emodin intake group was additionally markedly lower than that of emodin-free group (Figure 4A). However, the placental weights of blastocysts derived from both groups were comparable (data not shown). Finally, fetal weights were lower in the emodin intake (40 μM) than the untreated control group (Figure 4B). Our results collectively demonstrate that exposure of embryos to emodin reduces the potential of implantation and postimplantation development.

### 2.5. Apoptotic Effects of Emodin on Oocyte Maturation Status, Fertilization Rate, and Embryo Development during IVM

**Figure 5:**
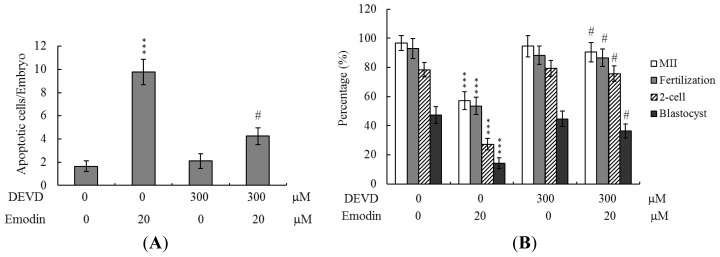

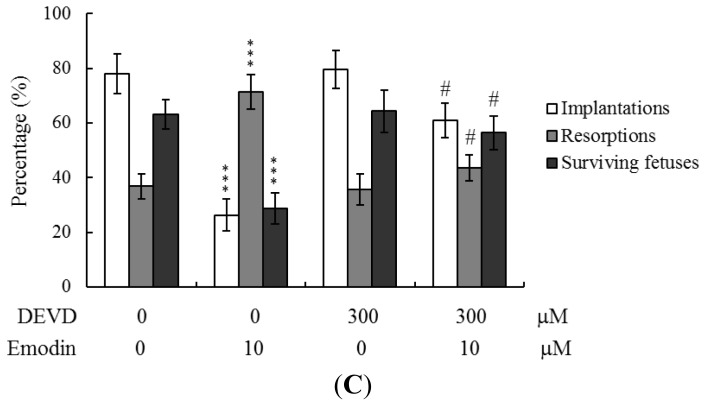
Effects of inhibition of apoptosis on embryo development in emodin- treated oocytes during IVM. Oocytes were collected from 21 day-old mice, cultured for 24 h in IVM medium alone or that containing 100 μM Ac-DEVD-cho and 20 μM emodin, fertilized *in vitro*, and transferred to *in vitro* culture (IVC) medium for *in vitro* development. (**A**) Apoptotic cells were examined at the blastocyst stage using TUNEL staining. (**B**) Oocyte maturation, *in vitro* fertilization, cleavage and blastocyst development rates were analyzed. (**C**) Implantation, resorption, and surviving fetuses were analyzed with the embryo transfer assay, as described for Figure 3. *** *p* < 0.001 *versus* the emodin-free group. # *p* < 0.001 *versus* the emodin-treated only group.

To further clarify the regulatory mechanisms of emodin, oocytes were pretreated with 100 μM Ac-DEVD-cho, a caspase-3 specific inhibitor, with the aim of preventing emodin-triggered embryo cell apoptosis during IVM of oocytes. Pretreatment with the caspase-3 inhibitor effectively prevented apoptosis of blastocysts derived from the oocyte group pretreated with 20 μM emodin (Figure 5A). Moreover, the caspase-3 inhibitor blocked emodin-triggered hazardous effects on oocyte maturation, fertilization rate, and sequential embryo development during IVM (Figure 5B), and additionally rescued reduction in postimplantation development potential following embryo transfer, leading to improvements in embryo implantation rate, fetal survival rate, and fetal development status (determined on the basis of fetal weight) (Figure 5C). These results strongly indicate that emodin regulation of oocyte development during IVM involves apoptotic processes.

## 3. Discussion

During the complex and precisely orchestrated process of oocyte maturation and early-stage embryonic development, chemical or physical injury can affect normal progression and lead to malformation or miscarriage of the embryo. Thus, it is crucial to ascertain the possible teratogenic effects of various agents on oocyte maturation and early-stage embryonic development. Emodin is a natural chemical compound in the root of rhubarb widely used in Chinese medicine. Recently, Wei and colleagues (2011) demonstrated that emodin not only induces apoptosis of cancer cells directly but also enhances the anticancer effects of gemcitabine in pancreatic cancer. Specifically, treatment with gemcitabine in combination with emodin efficiently inhibited tumor growth in mice inoculated with pancreatic tumor cells. The combination therapy induced a reduction in Akt and NF-κB activation and the Bcl-2/Bax ratio, and increase in caspase-9 and -3 activation, as well as cytochrome C release from the mitochondria to cytosol [23]. Thus, emodin appears to function as a chemopreventive and/or chemotherapeutic agent in different cancer types by decreasing cell viability, inhibiting cell proliferation and increasing apoptosis. A previous investigation by our group showed that co-incubation of embryos with emodin for 24 h triggers apoptosis in mouse blastocysts [12], in turn, suppressing cell viability. Measurement of embryo cell numbers of blastocysts using dual differential staining revealed emodin-induced cell loss and apoptosis in both ICM and TE cell populations [12]. In this report, we have examined the possible cytotoxic effects of emodin on oocyte maturation, fertilization, and sequential embryo development.

Oocyte maturation, fertilization, and embryonic development are complex processes during which chemical injury can lead to developmental problems or embryonic malformation. Previously, we reported that emodin induces apoptosis, impairment of blastocyst development from the morula and promotion of early-stage mouse blastocyst death [12]. In view of these findings, it is important to establish whether emodin has possible teratogenic effects. Here, we have shown for the first time that emodin inhibits mouse oocyte maturation, fertilization, and sequential embryonic development (Figure 1). Importantly, the emodin-pretreated oocyte group displayed a significant decrease in cell number and increased apoptosis (Figure 2A,B). In our experiments, cumulus-oocyte complexes (COCs) were isolated from female hybrid ICR mice (21 days old) injected with 5 IU human chorionic gonadotropin (hCG), 44 h prior to oocyte collection. Our coworkers additionally collected COCs from 42 day-old ICR mice. Importantly, no differences were observed in emodin-induced hazardous effects on mouse oocyte maturation and sequent embryonic development between oocytes from 21 and 42 day-old female mice (data not shown). The results collectively indicate that emodin treatment at the oocyte stage triggers both oocyte maturation injury and abnormal apoptosis of cells at the blastocyst stage, an important step in embryo implantation.

The TE arises from the trophoblast at the blastocyst stage, and develops into a sphere of epithelial cells surrounding the ICM and blastocoel. These cells contribute to the placenta, and are required for mammalian conceptus development [24]. Reduction of cells from the TE and/or ICM lineage leads to suppressed implantation and lower embryonic viability [25,26]. ICM and total blastocyst cell numbers are positively correlated with embryonic development during the embryo transfer assay [27]. In our experiments, application of emodin during oocyte maturation had no effect on the TE cell number of blastocysts, but induced a dramatic decrease in ICM and total (TE plus ICM) cell numbers (Figure 2). Our results imply that emodin treatment during IVM causes mortality and/or developmental delay in postimplantation mouse embryos via ICM cell death or decreased proliferation (Figures 2 and 3). Interestingly, blastocysts derived from emodin-treated oocytes appeared to undergo decreased implantation, and exhibited increased embryo resorption and lower fetal survival rate (Figure 3A), but comparable placental weights to the control group. TE cells of embryos play important roles in implantation and placental development. Our data showed that emodin does not exert a hazardous effect on TE cells of blastocysts, and consequently has no effect on placental development. Based on these findings, we propose that the decrease in ICM cell number induced by emodin during oocyte maturation is the major injurious factor leading to inhibition of embryonic development.

Emodin-pretreated oocytes displayed significantly decreased fertilization rates and cleavage to two-cell and blastocyst stages, compared to untreated control groups (Figure 1), indicating that emodin induces loss of fertilization and sequent embryonic development. Moreover, in an embryo transfer study, mouse blastocyst stage embryos derived from emodin-pretreated oocytes displayed lower implantation and higher resorption rates than control blastocyst-derived untreated oocytes (Figure 3A). Further experiments demonstrated significantly lower embryo implantation rates and fetal weights and higher resorption rate of blastocyst stage embryo transfer to mouse uterus in the emodin intake group, compared to those of the emodin-free control group (Figure 4A,B). Emodin has been shown to inhibit proliferation and induce apoptosis with an IC_50_ value of 20 μM in Jurkat cells [28]. Preliminary experiments by our group further revealed that emodin triggers mouse embryonic stem cell (ESC) apoptosis in a dose-dependent manner with an IC_50_ value of 12 μM, as determined from the MTT assay after 24 h of exposure (data not shown). In addition, our initial HPLC results disclosed serum emodin levels of about 8.7 μM in mice drinking water supplemented with 40 μM emodin over 4 days (data not shown). Our data showed that 10–20 μM emodin induces significant cytotoxic effects, *i.e.*, reduction in the rates of oocyte maturation, fertilization, and *in vitro* embryonic development. Accordingly, we propose that emodin suppresses oocyte maturation and *in vitro* fertilization and promotes early embryonic developmental injury at concentrations that may be attained via dietary intake. Clearly, emodin has potential hazardous effects on early-stage oocyte maturation and fertilization.

Emodin has been reported to trigger apoptosis of SCC-4 cells via mitochondria-dependent signaling pathways, and mitochondrial dysfunction as a result of Bcl-2 and Bax modulation, mitochondrial cytochrome c release and caspase activation [10]. Recent research in our laboratory demonstrated that pretreatment of cells with specific inhibitors against caspase-9 (Z-LEHD-FMK) and caspase-3 (Z-DEVD-FMK) effectively blocks apoptosis in mouse blastocysts [12]. In the current investigation, we showed that pretreatment of oocytes with emodin leads to decreased cell number, apoptosis, and delay in postimplantation development of blastocysts, compared to the control group. These injurious effects were prevented by pretreatment of oocytes with a caspase-3 inhibitor to suppress blastocyst apoptosis (Figure 5). Treatment with the caspase-3 inhibitor additionally rescued emodin-induced reduction in postimplantation development potential following embryo transfer, leading to improvements in embryo implantation rate, fetal survival rate, and fetal development status. Our results indicate that emodin triggers improper cell apoptotic processes in early-stage embryos, leading to loss of embryo cell numbers and suppression of post-implantation development, further supporting its role as a teratogen through apoptosis induction in early-stage cells. Accordingly, we conclude that developmental injury by emodin occurs via induction of apoptosis processes in oocyte maturation and early-stage embryos.

## 4. Experimental Section

### 4.1. Chemicals and Reagents

Dulbecco’s modified Eagle’s medium (DMEM), emodin, and pregnant mare serum gonadotropin (PMSG) were obtained from Sigma (St. Louis, MO, USA). Human chorionic gonadotropin (hCG) was purchased from Serono (NV Organon, Oss, The Netherlands). TUNEL *in situ* cell death detection kits were acquired from Roche (Mannheim, Germany), and CMRL-1066 medium from Gibco Life Technologies (Grand Island, NY, USA). Acetyl Asp-Glu-Val-Asp aldehyde (Ac-DEVD-cho) was from Calbiochem.

### 4.2. COC Collection and *in Vitro* Maturation (IVM)

ICR mice were acquired from the National Laboratory Animal Center (Taiwan). This research was approved by the Animal Research Ethics Board of Chung Yuan Christian University (Taiwan). All animals received humane care following Principles of Laboratory Animal Care (National Institutes of Health publication 85–23, revised 1996 [29]). Mice were maintained on breeder chow (Harlan Teklad chow) with food and water available *ad libitum*. Housing was provided in standard 28 cm × 16 cm × 11 cm (height) polypropylene cages with wire-grid tops, and maintained under a 12 h day/12 h night regimen. Cumulus-oocyte complexes (COCs) were obtained according to a previous protocol [14]. Briefly, COCs were isolated from female hybrid ICR mice (21 days old) injected with 5 IU human chorionic gonadotrophin (hCG) 44 h prior to oocyte collection. COCs were collected in HEPES-buffered α minimum essential medium (MEM) (containing 50 μg/mL Streptomycin sulfate, 75 μg/mL Penicillin G, and 5% fetal bovine serum) by gently puncturing visible antral follicles present on the ovary surface. Germinal vesicle stage oocytes containing an intact vestment of cumulus cells were collected and pooled in at least 10 animals. For oocyte maturation, one drop (~100 μL) of buffer (αMEM supplemented with 50 μg/mL Streptomycin, 75 μg/mL Penicillin G, 5% FBS and 50 mIU/mL recombinant human FSH) containing 10 COCs was added under oil in 35 mm culture dishes. COC maturation was analyzed following treatment with or without various concentrations of emodin (5, 10 or 20 μM) for 24 h under an atmosphere of 5% O_2_, 6% CO_2_ and balance of N_2_ at 37 °C.

### 4.3. Maturation Status Assessment

After *in vitro* maturation (IVM), COCs of each group were treated with 50 U/mL ovine hyaluronidase and gently pipetted for the removal of all cumulus cells. Denuded oocytes were collected, and washed with fresh medium, followed by phosphate-buffered saline (PBS). Oocytes were fixed in ethanol:glacial acetic acid (1:3) for 48 h, and stained with 1% aceto-orcein solution. Nuclear structures were visualized using phase-contrast microscopy.

### 4.4. *In Vivo* Maturation

For obtaining *in vivo* matured oocytes, 21 day-old mice were injected with 5 IU equine chorionic gonadotrophin (eCG) and 5 IU hCG, 61 and 13 h prior to fertilization, respectively. Mature ova were collected from the oviduct into HEPES-buffered in MEM medium.

### 4.5. Effects of Emodin Intake on Oocyte Maturation in an Animal Model

The effects of emodin on oocytes were analyzed in 21 day-old ICR virgin albino mice. Female mice were randomly divided into two groups of 20 animals each, and administered a standard diet with or without 10–40 μM emodin in drinking water for 4 days. COCs were collected by pre-treatment with 5 IU human chorionic gonadotrophin (hCG) for 44 h prior to oocyte collection, and analyzed for oocyte maturation, *in vitro* fertilization, and embryonic development.

### 4.6. *In Vitro* Fertilization

For *in vitro* fertilization, ova were washed twice in bicarbonate-buffered α-MEM medium (containing 50 mg/mL Streptomycin, 75 mg/mL Penicillin G and 3 mg/mL fatty acid free bovine serum albumin), and fertilized in the same medium with fresh sperm (obtained from a CBAB6F1 male donor). After incubation with sperm for 4.5 h, eggs were washed three times in potassium simplex optimized medium (KSOM) without amino acids in the presence of l-alanyl-l-glutamine (1.0 mM). Next, eggs were placed in 20 mL drops of KSOM under oil, and cultured overnight. During cleavage to the 2-cell stage, embryos were transferred to a fresh drop of KSOM under oil, and cultured for another 72 h. All fertilization steps and embryo culture were additionally carried out under 5% O_2_, 6% CO_2_ and balance of N_2_ at 37 °C.

### 4.7. Fertilization Assessment

For the examination of fertilization, ova were incubated with sperm for 4.5 h, followed by 3 h of culture in fresh medium. Zygotes were assessed for the presence of the male pronucleus with orcein staining, as described previously [14].

### 4.8. Cell Proliferation

Cell proliferation was analyzed by dual differential staining, which facilitated the counting of cell numbers in inner cell mass (ICM) and trophectoderm (TE) [25,30,31]. Blastocysts were incubated with 0.4% pronase in M_2_–BSA medium (M_2_ medium containing 0.1% bovine serum albumin) for the removal of zona pellucida. Denuded blastocysts were exposed to 1 mM trinitrobenzenesulfonic acid (TNBS) in BSA-free M_2_ medium containing 0.1% polyvinylpyrrolidone (PVP) at 4 °C for 30 min, and washed with M_2_[32]. Blastocysts were further treated with 30 μg/mL anti-dinitrophenol-BSA complex antibody in M_2_-BSA at 37 °C for 30 min, followed by M_2_ supplemented with 10% whole guinea pig serum as a source of complement, along with 20 μg/mL bisbenzimide and 10 μg/mL propidium iodide (PI) at 37 °C for 30 min. The immunolysed blastocysts were gently transferred to slides, and protected from light before observation. Under UV light, ICM cells (which take up bisbenzimidine but exclude PI) appeared blue, whereas TE cells (which take up both fluorochromes) appeared orange-red. Since multinucleated cells are not common in preimplantation embryos [33], the number of nuclei represent an accurate measurement of cell number.

### 4.9. TUNEL Assay of Blastocysts

For TUNEL staining, embryos were washed in emodin-free medium, fixed, permeabilized, and subjected to labeling using an *in situ* cell death detection kit (Roche Molecular Biochemicals, Mannheim, Germany), according to the manufacturer’s protocol. Photographic images were obtained with a fluorescence microscope under bright-field illumination.

### 4.10. Blastocyst Development Following Embryo Transfer

To determine the ability of expanded blastocysts to implant and develop *in vivo*, embryos generated were transferred to recipient mice. ICR females (6–8 week-old, white skin) were mated with vasectomized males (C57BL/6J; black skin; National Laboratory Animal Center, Taiwan) to produce pseudopregnant dams as recipients for embryo transfer. To ensure that all fetuses in pseudopregnant mice were derived from embryo transfer (white color) and not fertilization by C57BL/6J (black color), we examined skin color at day 18 post-coitus. To assess the impact of emodin on postimplantation growth *in vivo*, COCs were exposed to 0–20 μM emodin for 24 h, followed by fertilization and *in vitro* maturation to the blastocyst stage. Subsequently, 8 untreated control embryos were transferred to the left uterine horn, and 8 emodin-treated embryos to the right uterine horn in day 4 pseudopregnant mice. Forty surrogate mice were analyzed and killed on day 18 post-coitus, and the frequency of implantation calculated as the number of implantation sites per number of embryos transferred. The incidence rates of resorbed and surviving fetuses were calculated as number of fetuses per number of implantations, respectively. The weights of the surviving fetuses and placenta were measured immediately after dissection.

### 4.11. Statistical Analysis

Data were analyzed using one-way ANOVA and *t*-tests, and presented as means ± SD. Data were considered statistically significant at *p* <0.05.

## 5. Conclusions

Here, we have shown for the first time that emodin exerts injurious effects on oocyte maturation, fertilization, and embryonic development, which clearly suggesting that emodin is a risk factor for normal embryonic development that may reduce oocyte maturation in infertile couples. Further studies are required to determine the possible teratogenic actions of emodin on human oocyte maturation and embryogenesis.
